# A pragmatic approach to selective genetic testing in kidney transplant candidates

**DOI:** 10.3389/frtra.2023.1342471

**Published:** 2024-01-16

**Authors:** Pitchaphon Nissaisorakarn, Paul K. Fadakar, Kassem Safa, Andrew L. Lundquist, Cristian V. Riella, Leonardo V. Riella

**Affiliations:** ^1^ Division of Nephrology, Department of Medicine, Massachusetts General Hospital, Harvard Medical School, Boston, MA, United States; ^2^ Division of Nephrology, Department of Pediatrics, Massachusetts General Hospital, Harvard Medical School, Boston, MA, United States; ^3^ Division of Nephrology, Department of Medicine, Beth Israel Deaconess Medical Center, Harvard Medical School, Boston, MA, United States; ^4^ Center for Transplantation Sciences, Department of Surgery Massachusetts General Hospital, Boston, MA, United States

**Keywords:** donor testing, genetic testing, kidney transplantation, pathologic variants, recipient testing

## Abstract

**Introduction:**

Advances in the field of genetic testing have spurred its use in transplantation. Potential benefits of genetic testing in transplant nephrology include diagnosis, treatment, risk stratification of recurrent disease, and risk stratification in potential donors. However, it is unclear how to best apply genetic testing in this population to maximize its yield. We describe our transplant center's approach to selective genetic testing as part of kidney transplant candidate and donor evaluation.

**Methods:**

Transplant recipient candidates were tested if they had a history of ESRD at age <50, primary FSGS, complement-mediated or unknown etiology of kidney disease, or had a family history of kidney disease. Donors were tested if age <35, were related to their potential recipients with known genetic susceptibility or had a first-degree relative with a history of kidney disease of unknown etiology. A targeted NGS gene panel of 385 genes was used. Clinical implications and downstream effects were monitored.

**Results:**

Over 30% of recipients tested within the established criteria were positive for a pathogenic variant. The most common pathogenic variants were APOL1 high-risk genotypes as well as collagen 4-alpha-3, -4 and -5. Donor testing done according to our inclusion criteria resulted in about 12% yield. Positive test results in recipients helped with stratification of the risk of recurrent disease. Positive test results in potential donors guided informed decisions on when not to move forward with a donation.

**Discussion:**

Integrating targeted panel genetic testing into a kidney transplant clinic in conjunction with a selective criteria for testing donors and recipients ensured a reasonable diagnostic yield. The results had implications on clinical management, risk stratification and in some cases were instrumental in directing downstream changes including when to stop the evaluation process. Given the impact on management and transplant decisions, we advocate for the widespread use of genetic testing in selected individuals undergoing transplant evaluation and donation who meet pre-defined criteria.

## Introduction

Chronic kidney disease (CKD) has a prevalence of 8%–13% worldwide (1, 2). In the US, over 30 million people are affected by CKD (3). CKD is associated with high morbidity and mortality and represents a significant healthcare burden. One in four individuals with CKD report a family history of kidney disease suggesting a key role of genetics in the disease development (4, 5). To date, there are more than 500 mendelian disorders associated with kidney traits (6). Mendelian disorders are estimated to account for 10%–30% of adult chronic kidney disease and up to 70% in the pediatric population (7–9). Testing for these genetic conditions plays a major role in the diagnosis, prognostication and management of these diseases, though widespread use in the clinical setting was historically limited by cost, accessibility and the long turnaround time for results.

Major advances in sequencing techniques, expanded accessibility and lower cost of testing have heightened interest in integrating genetic testing into clinical practice. Due to the substantial role that genetics plays in nephrology, several studies have suggested the potential benefits of genetic testing in all aspects of nephrology including diagnosis, treatment, and in the field of kidney transplantation (7, 10, 11). However, the role of genetic testing in kidney transplantation is not well established. There are practice variations among centers, the yield of testing differs and how these results affect clinical management remains obscured. We aim to describe our transplant center's experience with genetic testing, specifically, how we selected candidates for testing in order to maximize the yield and describe the implications the results had on clinical management.

## Materials and methods

### Study subjects

**Figure 1:**
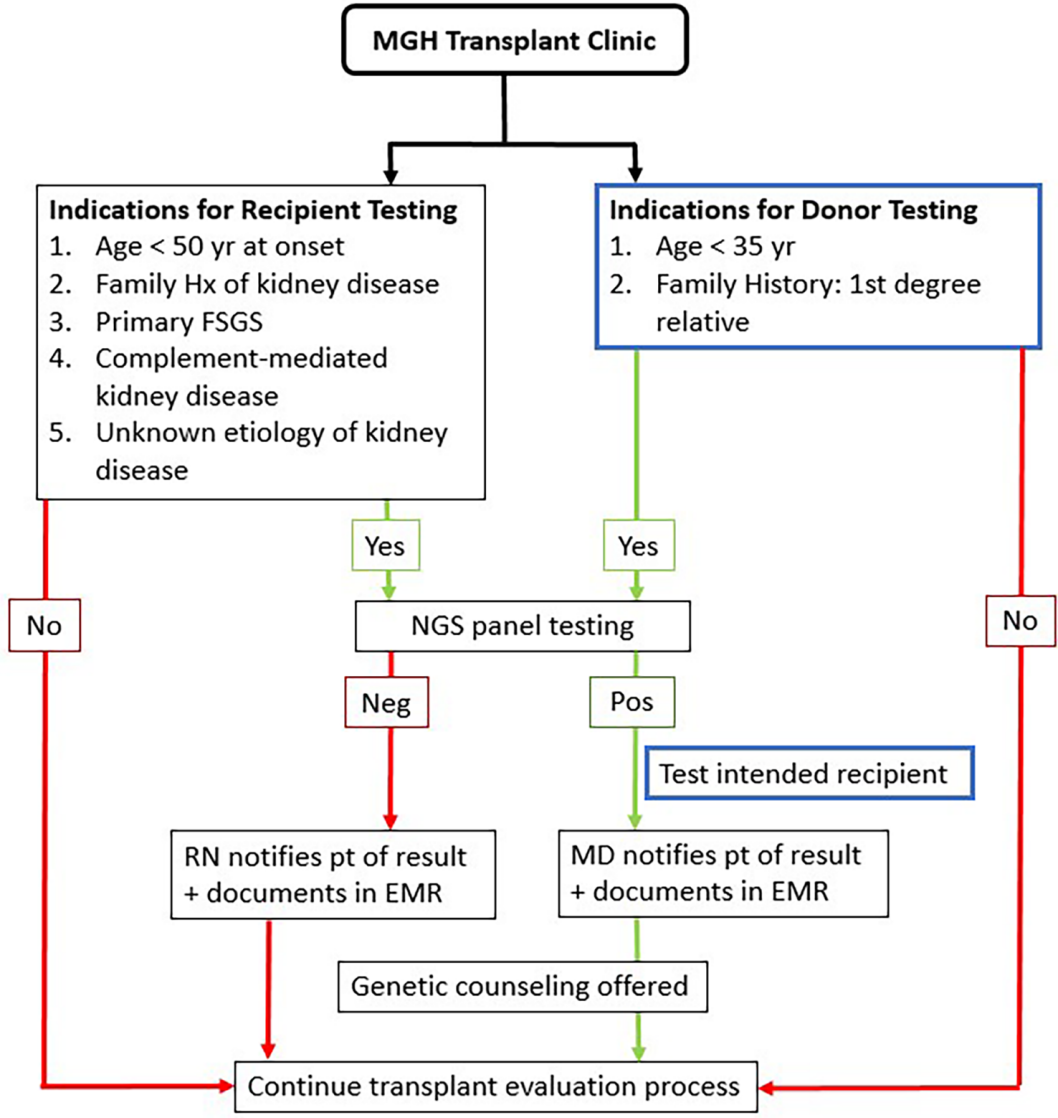
MGH Kidney transplant clinic genetic testing protocol.

An analysis of 83 tests performed with a 385 renal gene NGS panel (the RenasightTM test, Natera, San Carlos, CA, USA) was done. These tests were ordered by Nephrologists at Massachusetts General Hospital Transplant Nephrology Clinic between January 2021 and February 2022 according to pre-specified criteria (Figure 1).

Recipients were tested if they were under the age of fifty at the time of reaching ESRD, carried a diagnosis of primary FSGS, complement-mediated or unknown etiology of kidney disease, or had a family history of kidney disease. Donors were tested if they were younger than thirty-five, were related to their potential recipients with known genetic susceptibility or had a strong family history of a first-degree relative with kidney disease of unknown etiology.

Demographic information of the patients tested, including age, ethnicity, sex, transplant status, and testing indications specifying CKD stage and a limited set of CKD diagnoses was provided on the requisition form by the patient or physician. All patients or legal guardians provided informed consent for the performance of genetic testing and the data were de-identified prior to analysis.

The turnaround time for results was usually 3–6 weeks. Results were easily accessible to staff through the company's website and portal and results were also received via fax or mail. Patients were informed by our staff of the results either through telephone calls or secure messaging through electronic medical record. Patients were offered genetic counseling through the company if interested, free of charge. 10 out of 25 patients that tested positive and 14 out of 58 patients that tested negative had an appointment with a genetic counselor.

### Genetic panel

The broad renal genetic panel included 385 genes associated with cystic and tubulointerstitial disorders, glomerular disorders, complement-related kidney disorders, congenital anomalies of the kidney and urinary tract (CAKUT) and structural disorders, tubulopathy and tubular disorders, diabetic nephropathies, hypertension-related disorders, nephrolithiasis, and electrolyte abnormalities (7).

### Variant interpretation

Assessment of variants detected in the reportable region was based on the American College of Medical Genetics and Genomics guideline for sequence variant interpretation (12). Five categories were used to classify variants: pathogenic (P), likely pathogenic (LP), variants of uncertain significance (VUS), likely benign and benign. Variants were reported as follows: P and LP variants were reported. VUS findings were reported if requested by the provider but were not considered positive results. A monoallelic P/LP variant in an autosomal dominant (AD) or X-linked gene, and biallelic P/LP variants in an autosomal recessive (AR) gene were reported as positive findings. One P/LP variant in an AR gene was reported as carrier status. Clinical relevance of P/LP variants identified in genes associated with both AD and AR diseases was interpreted based on variant type, frequency, mechanism of disease, and previously reported clinical cases in literature. Regarding COL4A variants, heterozygous P/LP variants within the COL4A3 and COL4A4 were considered positive, as were heterozygous P/LP variants in COL4A5 in female patients (13).

## Results

### Demographics

Between January 2021 and December 2022, 1,233 recipient candidates were evaluated in our transplant clinic and 85 (7%) were recommended for genetic testing based on our inclusion criteria (Figure 1). Two patients did not have any results due to insufficient sample. Baseline characteristics of 83 potential recipients are shown in Table 1. About half of our patients were evaluated pre-dialysis. The majority of recipients were male (61.4%) while the majority of donors were female (58.8%). The mean age of recipients was 51 (±12.8). Donors' mean age was 36 (±11). More than half of our study population were Caucasian.

**Table 1:** Demographics of kidney transplant candidates and donors who underwent genetic testing.

Baseline clinical characteristics of patients		
	Transplant candidates (*n* = 83)	Donors (*n* = 17)
	Number of patients (percent)	
Gender—*n* (%)		
Female	32 (38.6)	10 (58.8)
Male	51 (61.4)	7 (41.2)
Age—*n* (%)		
0–18 year	0	0
19–29 year	5 (6.0)	5 (29.4)
30–49 year	34 (41.0)	10 (58.8)
50–69 year	36 (43.4)	2 (11.8)
≥70 year	8 (9.6)	0
Ethnicity—*n* (%)		
African American	18 (21.7)	2 (11.8)
Hispanic	10 (12)	0
Asian-American	11 (13.3)	3 (17.6)
Caucasian	42 (50.6)	11 (64.7)
Other	2 (2.4)	1 (5.9)
Diagnosis—*n* (%)		
ESRD	43 (51.8)	
CKD/Pre-dialysis	40 (48.2)	

### Diagnostic yield of genetic testing in recipients

**Figure 2:**
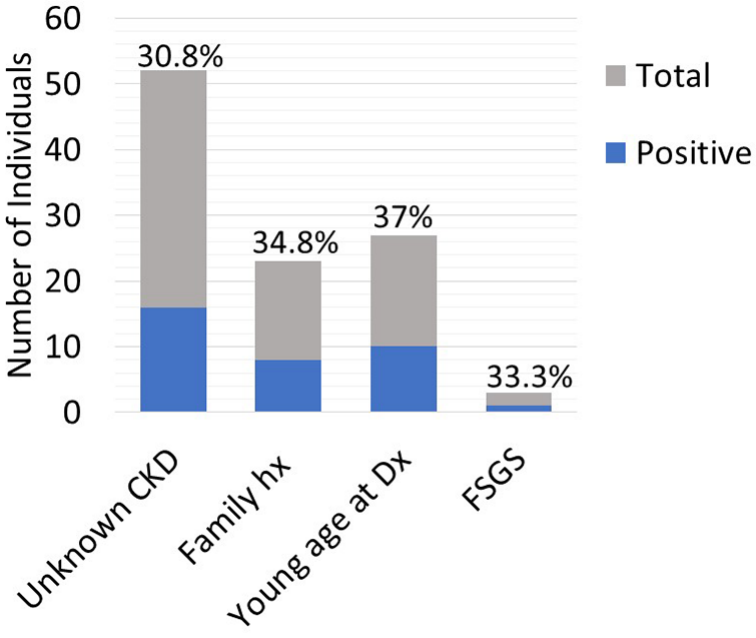
Diagnostic yield per indication for testing.

**Figure 3:**
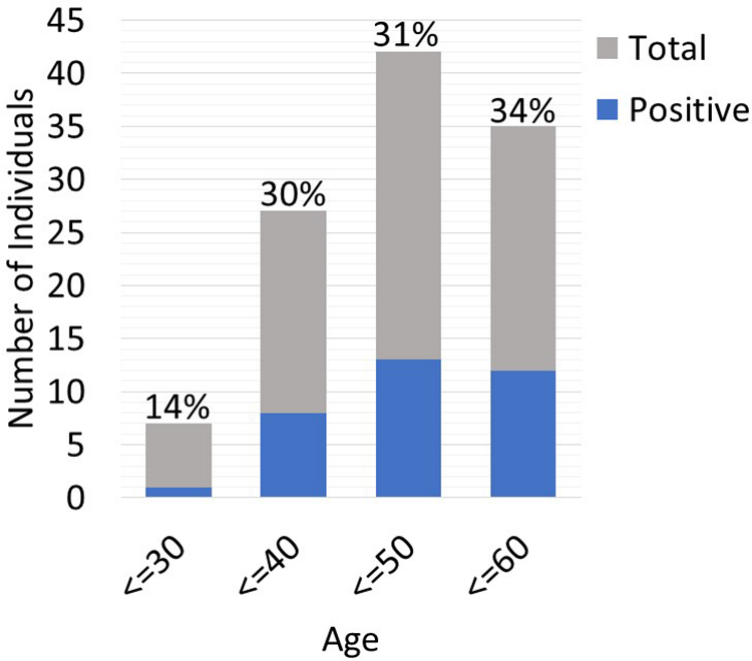
Diagnostic yield per age cut-off.

Twenty-five out of the 83 (30.1%) recipient candidates tested positive for pathogenic variants. Among our genetic testing indications, most were tested due to unknown etiology of renal disease (62.7%). Other indications for testing included young age at onset of disease (32.5%), strong family history (27.7%) and primary FSGS (3.6%). The diagnostic yield was similar across all indications and ranged from 30.8%–37% (Figure 2). Diagnostic yield per age cut off was comparable at around 30% when using the age cutoff of ≤40, ≤50, and ≤60 years old. However, the yield was considerably lower when the age cutoff of ≤30 was used (Figure 3). The most commonly found pathogenic variants were the presence of APOL1 high-risk genotypes (11.44%) followed by the COL4A group of genes (COL4A4—3 (12%), COL4A3—2 (8%), COL4A1—1(4%)). The remaining cases are as described in Table 2. Thirty-six (43.4%) patients were found to be carriers and all but one patient (98.8%) had genetic variants of unknown significance.

**Table 2:** Transplant candidates with positive pathogenic genetic testing.

	Race	Sex	Age	Clinical presentation	Family Hx	Biopsy results	Indication for testing	*Gene*	Variant	Inheritance	Zygosity
1	Caribbean African American	F	62	ESRD	None Reported	FSGS	Unclear etiology	*APOL1* *G1/G2*	c.[1024A>G;1152T>G] (p.[Ser342Gly;Ile384Met])	Complex	Heterozygous
									c.1164 1169del (p.Asn388_Tyr389del)		Heterozygous
2	Hispanic	F	50	ESRD	None Reported	Thin basement membrane with secondary FSGS	Young age at onset	*COL4A4*	c.3524G>C (p.Gly1175Ala)	AD&AR	Heterozygous
									c.2092G>A (p.Gly698Arg)		
3	Caucasian	F	67	CKD IV secondary to FSGS	Mother w/ CKD and hematuria	Collapsing FSGS	Primary FSGS	*COL4A4*	c.2644G>A (p.Gly882Se)	AD&AR	Heterozygous
4	African American	M	40	CKD V secondary to HTN	Paternal Aunt w/ ESRD s/p transplant; Grandmother on HD	Chronic changes, Glomerulosclerosis and IFTA	Unclear etiology and family history	*APOL1* *G1/G2*	c.[1024A>G;1152T>G] (p.[Ser342Gly;Ile384Met])	Complex	Heterozygous
									c.1164_1169del (p.Asn388_Tyr389del)		Heterozygous
5	Caucasian	M	59	ESRD; hypertension	Yes, not specified	None	Unclear etiology	*UMOD*	c.278_289delinsCCGCCTCCT (p.Val93_Gly97delinsAlaAlaSerCys)	AD	Heterozygous
6	African American	M	60	ESRD	Paternal uncle w/ ESRD *Sister, maternal aunt w/ SLE* Parents w/ HTN	None	Unclear etiology	*APOL1* *G2/G2*	APOL1 c.1164_1169del (p.Asn388_Tyr389del) (G2 allele)	Complex	Homozygous
7	Caucasian	F	39	CKD5, nephrolithiasis	Father w/ CKD, mother and brother w/ nephrolithiasis	None	Unclear etiology and young age at onset	*COL4A1*	COL4A1 c.2317G>A (p.Gly773Arg)	AD	Heterozygous
8	African American	M	33	ESRD, FSGS	None reported	FSGS and chronic changes	Unclear etiology and young age at onset	*APOL1* *G1/G2*	c.[1024A>G;1152T>G] (p.[Ser342Gly;Ile384Met]) (G1 allele)	Complex	Homozygous
									TTR c.424G>A (p.Val142Ile)	AD	Heterozygous
9	Caucasian	F	34	ESRD secondary to chronic tubulointerstitial nephritis, associated with nephronopthisis	Aunt w/ HTN Cousin s/p kidney transplant	Chronic changes, secondary glomerulosclerosis	Young age at onset	*NPHP1*	c.555del (p.Lys185Asnfs*7)	AR	Homozygous
10	African American	M	50	ESRD secondary to IgA nephropathy	Sister w/ CKD	IgA nephropathy	Family history and young age at onset	*PROKR2*	c.278_289delinsCCGCCTCCT (p.Val93_Gly97delinsAlaAlaSerCys)	AD	Heterozygous
11	Caucasian	F	62	ESRD, HTN, DM	Father w/ GN Paternal grandmother, paternal great aunt and paternal cousin w/ CKD	None	Unclear etiology and family history	*UMOD*	c.278_289delinsCCGCCTCCT (p.Val93_Gly97delinsAlaAlaSerCys)	AD	Heterozygous
12	Caucasian	M	64	ESRD, DM	Mother with PKD	Secondary FSGS	Family history	*COL4A3*	c.2452G>A (p.Gly818Arg)	AD&AR	Heterozygous
13	Hispanic	F	53	ESRD	Brother and daughter with CKD, half-brother s/p renal transplant	Collapsing glomerulopathy	Primary FSGS	*APOL1* *G1/G1*	c.[1024A>G-1152T>G] (p.[Ser342Gly;Ile384Met])	Complex	Homozygous
14	African American	M	56	CKD stage 5 due to secondary FSGS and thin basement membrane	Father w/ HTN and CKD Brother with ESRD, another brother w/ HTN and CKD	Chronic changes w/ diffuse global and segmental glomerulosclerosis and severe vascular sclerosis	Family history	*APOL1* *G1/G2*	c.[1024A>G-1152T>G] (p.[Ser342Gly;Ile384Met])	Complex	Heterozygous
									c.1164_1169del (p.Asn388_Tyr389del)		
								*COL4A4*	c.3734G>T (p.Gly1245Val)	AD&AR	Heterozygous
15	Caucasian	F	68	CKD IV due to PKD	Daughter w/ thin basement membrane disease	None	Family history	*COL4A3* *G1/G2*	c.[1024A>G;1152T>G] (p.[Ser342Gly;Ile384Met])	Complex	Heterozygous
									c.1164_1169del (p.Asn388_Tyr389del)		Heterozygous
16	African American	F	60	ESRD, HTN, RCC s/p R nephrectomy	Father and sister w/ ESRD on HD	None	Family history and unclear etiology	*APOL1* *G1/G2*	c.[1024A>G;1152T>G] (p.[Ser342Gly;Ile384Met])	Complex	Heterozygous
									c.1164_1169del (p.Asn388_Tyr389del)		Heterozygous
17	African American	F	31	ESRD due to collapsing FSGS	None reported	Collapsing FSGS	Young age at onset, FSGS	*APOL1* *G1/G1*	c.[1024A>G;1152T>G} (p.[Ser342Gly;Ile384Met])	Complex	Homozygous
18	African American	M	38	ESRD on HD, presumed due to HTN	None reported	None	Young age at onset and unclear etiology	*APOL1* *G1/G2*	c.[1024A>G;1152T>G] (0.[Ser342Gly;Ile384Met])	Complex	Heterozygous
									c.1164_1169del (p.Asn388_Tyr389del)	Complex	Heterozygous
19	Caucasian	M	48	CKD 5 presumed due to DM, obesity	Father w/ CKD	None	Young age at onset, unclear etiology and family history	*WT1*	c.320G>A (p.Trp107)	AD	Heterozygous
20	Caucasian	M	53	CKD from failing transplant, RCC of native kidney	None reported	None	Unclear etiology	*APOL1 G1/G1*	c.[1024A>G;1152T>G] (p.[Ser342Gly;Ile384Met])	Complex	Homozygous
								*SLC3A1*	Duplication of Exons 5-9	AD&AR	Not applicable
21	Caucasian	F	60	CKD IV due to FSGS and thin BM	Daughter w/ Alports disease	Secondary FSGS and thin basement membrane	Primary FSGS,	*COL4A4*	c.2529 2537delinsAT (p.Tyr844Leufs*23)	AD&AR	Homozygous
22	Caucasian	M	48	CKD IV secondary to nephrolithiasis, Severe bilateral medullary nephrocalcinosis	Father w/ nephrolithiasis	None	Young age at onset and unclear etiology	*SLC4A1*	c.1825G>A (p.Gly609Arg)	AD/AR	Heterozygous
23	African American	M	29	CKD IV secondary to collapsing FSGS, obesity, HTN	None reported	Collapsing FSGS	Young age at onset and unclear etiology	*APOL1 G1/G1*	c.[1024A>G;1152T>G] (p.[Ser342Gly;Ile384Met])	Complex	Homozygous
24	Caucasian	M	74	ESRD secondary to cystic kidney disease and congenital single kidney	Sibling w/ nephrolithiasis and cysts, child w/ CKD	None	Family history and unclear etiology	*GATA3*	c.708dup (p.Ser237Ginfs*67)	AD	Heterozygous
25	Caucasian	M	37	ESRD secondary to failing kidney transplant, Original kidney disease unclear	None reported	Advanced antibody and T-cell mediated rejection	Young age at onset and unclear etiology	*SMARCAL1*	c.2114C>T (p.Thr70511e)	AR	Heterozygous
									c.2542G>T (p.Glu848*)	AR	Heterozygous

### Diagnostic yield of genetic testing in potential donors

Seventeen potential donors underwent testing as part of their donor work-up. They were tested if they met our pre-specified criteria (Figure 1). Two potential donors (11.8%) were found to be positive for *COL4A4.* Both donors were tested because they were first-degree relatives of the intended recipients (#1 were siblings, #2 was an identical twin). Potential donor#1 had the same pathogenic variant as the potential recipient [*COL4A4* c.4522G > C (p.Gly1508Arg)]. The potential recipient for donor#2 (her identical twin) had not undergone genetic testing as the underlying cause of her end-stage kidney disease was proliferative glomerulonephritis with monoclonal IgG deposits, a relatively new entity that is less well known and so far has not shown a genetic predisposition.

### Clinical implications and downstream effects

Recipient testing was helpful for prognostication and risk-stratifying for disease recurrence posttransplant. Once a recipient was found to have a genetic variant, this set into motion a series of clinical events. Our providers communicated the results to the recipients, conveyed to them the risk of recurrence post transplant, offered genetic counseling and recommended testing for immediate family members. As examples, potential kidney transplant recipient with presumed primary FSGS who tested positive for genetic predisposition such as having 2 high-risk APOL1 variants or a collagen-associated mutation, were deemed very low-likelihood of recurrence compared to 30%–50% recurrence risk in those without a genetic mutation (Table 2).

On the donor side, as demonstrated in the cases described above, the two positive test results resulted in termination of the donor evaluation process for these two potential donors. For those donors that tested negative, this allowed them to proceed with the rest of the donor evaluation after providing reassurance to both the providers and potential donors that the risk to the donor was low and acceptable. This was highlighted in a donor recipient pair: the brother of a recipient with FSGS and homozygous high-risk APOL-1 alleles wished to donate. Donor testing showed that he was heterozygote for the APOL-1 high risk allele and he was cleared to donate. An exception to this was a 33-year-old female who wished to donate to her dad who has polycystic kidney disease (PKD). She had 2 small cysts on imaging. She tested negative for any pathologic variants but had PKD VUS (*PKD2*). We tested her dad and both had the same PKD VUS (*PKD2*). In this situation, we declined her for donation.

## Discussion

By predefining specific criteria for genetic testing in potential candidates and donors going through the kidney transplant evaluation process, we were able to achieve a diagnostic yield of 30.1% and 11.8%, respectively. The results of genetic testing impacted the ensuing steps in the evaluation process. Our results confirm that genetic testing when applied to select individuals resulted in high yield and culminated in meaningful changes in clinical management.

Recent advances in the field of genetics and its myriad of benefits have spurred interest in how this can best be applied to the field of nephrology. Groopman et al. applied whole exome sequencing (WES) in a combined cohort of more than 3,000 patients with CKD, with a diverse array of pathology and no preselection for cases concerning for genetic disease, and found diagnostic mutations in 9%. Connaughton et al. performed WES in 138 adults with CKD and was able to identify a molecular genetic diagnosis in 37% of patients. The yield was especially high in those with extrarenal manifestations of disease (69%) and in those with positive family history (36%).

Genetic testing in the kidney transplant setting is less studied. In a cohort of 142 patients on the waitlist for a kidney transplant at a single center, renal gene panel testing of 209 genes was done in 57 patients who had an undetermined cause of ESRD. A genetic cause of ESRD was established in 12% (14). In another report, targeted gene-panel testing done in patients younger than 40 years of age with nephropathy of unknown origin and end-stage renal disease on the wait list for a transplant (656 patients), a high proportion of patients [15 of 81 (19%)] were found to have pathogenic or likely pathogenic variants (15). Among the most common pathogenic variants were COL4A3, COL4A4, COL4A5, and other genes that are implicated in focal segmental glomerulosclerosis. The authors suggested that genetic testing in a pre-selected cohort may be useful.

It has been estimated that around 15% of transplant recipients have an unknown etiology of ESRD which can potentially be hereditary (16, 17). Thus, there is a gap in identifying previously undiagnosed or misdiagnosed native kidney disease and ensuring that potential donors have as much information as possible to aid in their decision-making process, particularly when they are a close relative to the recipient.

Our study aimed at integrating genetic testing into the workflow of an outpatient transplant nephrology clinic. 7% of patients undergoing transplant evaluation were tested according to our criteria. The diagnostic yield from this cohort was 30.1% which is comparable to previous studies (24%–43%) (8, 10, 18, 19). If an unbiased approach to genetic testing is taken, close to 9% of CKD patients with an unknown cause were found to have a pathogenic variant that led to kidney disease (7). Our yield of more than 30% indicates the benefit of selective testing of potential recipients, allowing us to maximize the yield of genetic testing in a patient population that has been less studied without incurring unnecessary cost to the evaluation process (20–22). Advances in genetics also allow us to rapidly screen and identify single gene mutations in patients with chronic kidney disease. This has led the way in risk-stratifying recurrent diseases based upon disease mechanism. For example, those with a monogenic cause of FSGS such as the presence of two high-risk APOL1 variants or type 4 collagen mutations (non-circulating factor) have minimal risks of recurrence post-transplant. Whereas in cases in which no genetic mutations have been identified, the risk of recurrence is variable. A subset of these patients will be at high risk for recurrence post-transplant, in some as high as 80% (23). Those that have rapid recurrence of FSGS are thought to be due to a yet to be identified “circulating factor” (24). In certain diseases with multiple causative mutations, such as atypical HUS, determining the specific causative mutation can allow for stratification of the patient based on the known risk of recurrence (i.e., low for MCP mutations vs. high for CFH mutation). Differentiating those with monogenic variations using available gene panels, will allow for a more streamlined process of recipient evaluation and to be able to provide reassurance in those instances where there is a low likelihood of recurrence post-transplant.

Donor evaluation is another aspect of kidney transplantation in which genetic testing can be of significant benefit. Kidney donors are at higher risk of developing hypertension, ESRD, and subsequently, higher morbidity and mortality from cardiovascular causes (25–27). Hence potential donors must go through meticulous work-up to minimize post-donation risks. The risk of developing ESRD is higher in donors who are related to their recipient even in instances where no monogenetic cause of kidney disease has been identified (16, 17). In our cohort, of the 17 donors that were tested, 2 had a positive genetic test. The result of donor testing is instrumental in guiding next steps in the work-up. In a donor who tests positive for a type 4 collagen mutation, the evaluation is stopped due to risk for kidney disease themselves either at baseline or post-donation. However, in other instances, such as a donor who tests positive for an APOL1 mutation, the path forward is less clear. Not all individuals carrying two APOL1 high-risk variants develop kidney disease and “second-hits” are required for deterioration in kidney function. Currently, there is a lack of robust data on the post-donation risk of ESRD in potential LKDs carrying two APOL1 high-risk variants. Ongoing studies such as APOLLO will help clarify this risk. Meanwhile, transplant centers have taken different approaches. In this center, an older donor with 2 APOL1 high-risk variants may be considered for donation after informed consent. Our cohort also highlights the need for candidate testing. Related potential donors of patients found to have the same mutation or suspicious VUS as the patient, are generally ruled out. This is especially helpful in patients who don't have the typical phenotype of the disease. Limitations of our study include small sample size, single-center, and short follow-up period. In addition, not all genetic causes may have been identified in a genetic panel testing. and whole exome sequencing may be indicated as a second step for patients with strong family history. Close collaboration with a genetic kidney team is crucial to guide next steps and also help interpret VUSs, in particular those autosomal dominants with a possible relevance to the phenotype. A recurrent meeting every month to review the results in a team-based approach would be recommended for centers systemically pursuing genetic testing in transplantation. The generalizability of this study may be limited, especially in smaller centers with less resources. Differences in insurance coverage and cost of testing in other resource-limited areas, may also be prohibitive of more widespread testing.

Advances in the field of genetics have opened up new horizons including in the field of kidney transplantation. Immeasurable benefits await those who can expertly wield these recent developments. An impediment in doing so comes in the form of how to best utilize it in the regular clinical setting. We demonstrate a pilot approach that was able to maximize the yield, leading the way for bigger projects down the road. Large scale projects are needed to further study the role of genetic testing in kidney transplantation.

## Conclusions

By establishing a genetic testing protocol for recipients and donors in our kidney transplant clinic, we were able to provide testing at a reasonable yield and the results complemented the evaluation process going forward. Given the decreased cost of testing, expanded accessibility, faster turnaround time and explosive growth of available literature in the field, minimal barriers remain to prevent widespread use of genetic testing in the kidney transplant field. With acceptable cost and burgeoning benefits of testing, we advocate for widespread use of genetic testing in clinical practice in kidney transplantation.

## Data Availability

The original contributions presented in the study are included in the article/Supplementary Material, further inquiries can be directed to the corresponding author.
